# Prioritizing Barriers and Facilitators to PrEP Uptake Among Black Cisgender Women: Key Factors Identified Through Nominal Group Technique

**DOI:** 10.3390/ijerph23050571

**Published:** 2026-04-28

**Authors:** Amber I. Sophus, Alex Dubov, Aaliyah Gray, Chika C. Chuku, Mandy J. Hill, Jamila K. Stockman, Jason W. Mitchell

**Affiliations:** 1 Department of Health, Behavior, and Society, Kate Marmion School of Public Health, University of Texas at San Antonio, San Antonio, TX 78229, USA; 2 Center for Interdisciplinary Research on AIDS, Yale University, New Haven, CT 06510, USA; 3 School of Public Health, Loma Linda University, Loma Linda, CA 92350, USA; 4 Center for Women’s and Gender Studies, College of Arts, Science, and Education, Florida International University, Miami, FL 33199, USA; 5 Department of Health Promotion and Disease Prevention, Robert Stempel College of Public Health & Social Work, Florida International University, Miami, FL 33199, USA; 6 Department of Population Health & Health Disparities, School of Public and Population Health, University of Texas Medical Branch at Galveston, Galveston, TX 77555, USA; 7 Division of Infectious Diseases and Global Public Health, Department of Medicine, University of California, San Diego, La Jolla, CA 92093, USA

**Keywords:** nominal group technique, focus group, women and PrEP, HIV prevention, pre-exposure prophylaxis, Black women, healthcare access

## Abstract

**Highlights:**

**Public health relevance—How does this work relate to a public health issue?**

- Black women experience disproportionate HIV incidence and prevalence rates in the United States but remain underrepresented among pre-exposure prophylaxis (PrEP) users.
- This study identifies and prioritizes key barriers and facilitators influencing Black women’s potential PrEP use in the US South.

**Public health significance—Why is this work of significance to public health?**

- Using Nominal Group Technique, the study highlights the most salient concerns affecting PrEP uptake among Black women, including medication side effects, drug interactions, and lack of health insurance coverage.
- Findings emphasize the importance of culturally responsive care, particularly the role of race- and gender-concordant providers in supporting PrEP engagement among Black women.

**Public health implications—What are the key implications or messages for practitioners, policy makers, and/or researchers in public health?**

- Community-centered strategies, including culturally tailored outreach, trusted providers, and targeted education campaigns, may improve PrEP awareness, appeal, and uptake.

**Abstract:**

Existing research has identified multi-level barriers and facilitators associated with pre-exposure prophylaxis (PrEP) uptake among Black women (BW); little is known about how BW prioritize these factors. In this study, Nominal Group Technique (NGT) was used to identify and prioritize key barriers and facilitators influencing BW’s potential use of PrEP. NGT sessions were conducted in two online focus groups with adult BW without HIV (N = 14). Participants ranked 16 barriers and 16 facilitators related to PrEP, by importance from 1 to 16, followed by a group discussion to review rankings, clarify reasons, and consolidate options. Participants also offered suggestions to enhance PrEP uptake for BW. Top concerns about PrEP were (1) side effects; (2) potential interactions with other medications; and (3) lack of health insurance coverage for PrEP. Key factors influencing PrEP use included: (1) discussing PrEP with a doctor of the same race; (2) discussing PrEP with a doctor of the same gender; and (3) receiving regular text or email reminders to take PrEP. Participants emphasized the importance of having race- and gender-concordant providers, which significantly influenced their consideration of PrEP. Using NGT as a qualitative exploratory method, this study underscores the importance of addressing key barriers and facilitators to PrEP uptake among BW.

## 1. Introduction

Black cisgender women (BW) account for over 50% of those diagnosed with HIV among women [1,2], despite representing only 13% of the US female population [2,3]. This disparity is especially pronounced in the South, where HIV incidence rates among BW aged 13 and older are the highest [2]. Encouraging BW to use and adhere to pre-exposure prophylaxis (PrEP) offers a viable strategy to reach zero new HIV cases and HIV transmissions [4].

PrEP, a proven preventative antiretroviral medication, greatly reduces the likelihood of acquiring HIV by over 95% among individuals with a negative serostatus. Increasing PrEP use among populations vulnerable to acquiring HIV is a central goal of the US “Ending the HIV Epidemic” initiative [5]. Unlike other prevention methods (e.g., condoms), which may require partner negotiations, PrEP provides BW the autonomy and empowerment to make independent decisions about their sexual health and HIV prevention. However, BW remain vastly underrepresented among PrEP users compared to women of other racial groups [1,6,7,8,9,10,11,12], and face unique barriers along the PrEP care continuum [12,13,14,15,16,17,18,19,20]. These barriers include limited knowledge and awareness of PrEP [21,22,23,24,25,26], low perceived HIV vulnerability or exposure, lack of health insurance [20,21,24,27,28,29,30,31,32,33,34,35,36,37], unreliable or inadequate transportation to access PrEP [38,39,40], insufficient communication between providers and patients about sexual behaviors that may increase their need for HIV prevention and/or PrEP [6,41,42], medical mistrust [43,44,45,46,47], PrEP-related stigma [41,48,49], and syndemic conditions (e.g., racism, poverty, low education, behavioral health issues, partner violence, homelessness) [50,51,52]. Additionally, limited PrEP marketing targeting BW remains a societal and individual-level barrier to PrEP uptake [16,19]. Despite these challenges, factors that may encourage PrEP use among BW (i.e., facilitators) include heightened perception of one’s vulnerability to acquiring HIV [6,53], concerns about a partner’s sexual behaviors [54], and knowing others who use PrEP [54,55,56]. Learning about PrEP from trusted sources (e.g., a friend living with HIV, primary healthcare provider, and/or other BW) [37,53,55] and having positive interactions with PrEP providers [37,53,54,55,57] also facilitates PrEP engagement among BW. Healthcare providers play a key role in providing PrEP information and access to prescriptions [6,22,24,35,40,41,57]. To determine PrEP eligibility, in many cases, an individual must be willing to meet with a healthcare provider (either in-person or online) and openly discuss behaviors that may increase their vulnerability or exposure to HIV (i.e., sexual practices, substance use, etc.). As such, the patient–provider relationship may be crucial for PrEP education, access, and uptake among populations vulnerable to HIV acquisition.

While existing research has identified multi-level barriers and facilitators associated with PrEP uptake among BW, these studies have primarily focused on describing what factors influence PrEP engagement rather than determining their relative importance in decision-making. As a result, there is limited understanding of which modifiable barriers and facilitators are most influential in shaping PrEP uptake intention, and which should be prioritized in intervention development. This gap is important to note since effective PrEP interventions require prioritization of key actionable determinants, particularly in the context of limited resources and complex, multi-level barriers to uptake. Thus, identifying which modifiable barriers and facilitators are most important to BW can provide actionable insights for prioritizing intervention targets to improve engagement along the PrEP care continuum. The Nominal Group Technique (NGT) [58,59,60,61,62] is particularly well-suited for this purpose.

The NGT is a consensus method [58] ideal for generating quantitative rankings (real-time consensus scoring) while also collecting in-depth qualitative information within a short timeframe. To our knowledge, no study has used NGT in an online format to identify potential PrEP program targets among BW. This study used NGT in an online format to identify which modifiable barriers and facilitators hold the most “value” in BW’s PrEP decision-making process.

## 2. Materials and Methods

NGT is a structured, multi-step method that integrates both individual and group-based data collection. NGT prompts participants to first generate ideas independently, complete an initial ranking exercise, followed by a structured group discussion in which responses are clarified and contextualized, and, finally, an opportunity to revise rankings. In this way, NGT includes a facilitated group discussion component similar to a focus group; however, this discussion represents only one step within a broader process that prioritizes individual input and quantitative ranking. NGT is commonly used in complex decision-making scenarios [58,59,60,61] and is adaptable across different settings and methodologies [59,62]. This approach is well-suited to the current study, which seeks to identify and prioritize factors influencing hypothetical PrEP decision-making. The initial independent ranking ensures that individual perspectives are captured without influence from other participants, while the group discussion allows participants to reflect on and refine their reasoning. The final ranked outputs therefore represent aggregated individual priorities rather than consensus derived solely from group interaction. Compared to traditional focus groups, which rely primarily on open-ended discussion, NGT provides a more structured approach to systematically identify and prioritize factors relevant to decision-making, ensures equal participation, and reduces researcher bias by limiting dominant voices in the conversation [60]. Other consensus methods, such as the Delphi technique, rely on “interactions between group (called panel) members via questionnaires rather than face-to-face communication” [58,59,63]. However, the Delphi technique is often used to develop guidelines rather than explore ideas in relation to a problem or question [58,63], has a lower response rate of success, and cannot be completed within a short timeframe [59,64].

In this study, the terms ‘focus group’ or ‘focus group discussion’ are used to describe the structured group discussion component of the NGT process rather than a separate qualitative method.

### 2.1. Study Participants

Two focus groups (FG1 and FG2) were held between February and March 2024, each with 6–7 unique participants. BW were recruited through advertisements on Facebook and Instagram to participate in a one-time, online NGT FG session. Individuals were eligible to participate if they self-reported and met all of the following inclusion criteria: (1) 18 years of age or older; (2) cisgender woman; (3) Black (i.e., African American, Caribbean American, etc.); (4) HIV-negative or unknown serostatus; (5) lived in a state within the US South; (6) owned a web-connected device with a video camera; and (7) fluent in English. To ensure that the factors listed in the NGT were understandable across different literacy levels, and to account for BW facing socioeconomic challenges, who are often underrepresented in PrEP research and have higher vulnerability to HIV acquisition, eligible participants also had to meet at least one of the following criteria: (8a) educational attainment of less than a bachelor’s degree; (8b) annual income < $50,000; (8c) not employed full-time. This approach worked to improve the applicability of the study findings towards developing inclusive and effective PrEP programming for BW, particularly those who may face additional barriers to PrEP access and uptake.

### 2.2. Study Procedures

The study protocol was approved by the Institutional Review Board at Florida International University (IRB-23-0283-AM01). Qualtrics, a HIPAA-compliant, web-based survey program (Qualtrics, Provo, UT, USA), hosted the study informational landing webpage. The study webpage provided interested individuals access to complete the e-consent form followed by the eligibility screener. Consented and eligible individuals were then evaluated against fraud deterrent and detection procedures [65] before being contacted (via email and/or phone call) to participate in a one-time, online FG via Zoom.us (video conferencing platform; HIPAA-compliant license hosted on a university server) for approximately 90 min. Individuals who confirmed and participated in the online FG were enrolled in the study. Recommendations and best practices for conducting NGT as focus groups [60,61,62] in a virtual setting [62,66] were followed. NGT rankings were conducted using Google Sheets. A trained facilitator (who also identified as a cisgender Black woman) led each session using a semi-structured focus group guide (see Supplementary Materials), while a co-facilitator took notes and provided technical support (if needed). To ensure participants felt comfortable and safe during each session, participants also provided verbal consent to be audio- and video-recorded before the session began. Those who completed the FG received a $75 Amazon e-gift card via email. FG recordings were transcribed using a third-party transcription service (https://www.rev.com), with all identifiers removed before analysis.

### 2.3. Nominal Group Technique Process

Items selected for the NGT were extracted from previously published studies and systematic reviews that focused on PrEP decision-making, potential PrEP uptake, PrEP engagement, and PrEP delivery preferences among Black women in the US [6,7,16,36,40,43,46,54,56,65,67,68,69,70,71,72,73,74,75,76,77,78]. Items were selected based on their relevance as modifiable barriers and facilitators. Where overlapping concepts were identified in the literature, they were consolidated into distinct, non-redundant items for inclusion in the NGT. Items were intentionally balanced to maintain equivalence and to reduce participant burden during the NGT ranking process, resulting in 16 modifiable barriers and 16 facilitators related to PrEP decision-making specific to Black women.

**Figure 1:**
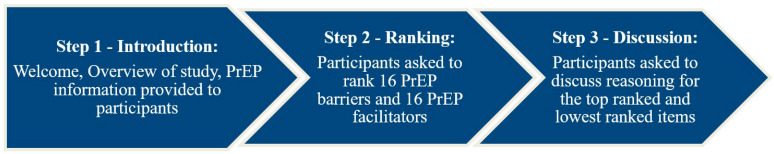
Steps in the nominal group technique.

The NGT process consisted of three steps (see Figure 1). Step 1. After a welcome from the facilitator, participants received an overview of the study’s purpose, procedures, and the focus group (FG) process (i.e., the facilitated group discussion following NGT rankings). The facilitator used a PowerPoint presentation to explain PrEP, including its purpose, effectiveness, potential side effects, available PrEP modalities, and the process for obtaining it. Prior PrEP awareness was not formally assessed to minimize priming and framing bias that could influence how participants interpreted and ranked the standardized items. Instead, all participants received the same standardized PrEP information and were given an opportunity to ask clarifying questions before beginning the ranking exercise.

Step 2. Participants were given a link to a Google Sheet via Zoom chat to rank 16 modifiable PrEP-related barriers and 16 facilitators from 1 (most important) to 16 (least important). Each participant worked on an individual sheet displaying the options in one column and a drop-down list for ranking in the adjacent column. Once rankings were submitted, a summary sheet automatically displayed the aggregated group rankings and anonymous individual scores for the facilitator. In line with prior research [79], we omitted the initial “silent generation of ideas” step as the literature review provided sufficient background on the barriers and facilitators of PrEP use among BW [6,7,16,36,40,43,46,54,56,65,68,69,70,71,72,73,74,75,76], including preferences for receiving PrEP and/or PrEP delivery [76,77,78].

Step 3. The group reviewed the ranking summary, focusing only on the top 3 highest and lowest priority items. Participants discussed their reasoning for these rankings, providing context without pressure to reach a consensus or change their individual scores. During the discussion, participants also suggested barriers or facilitators to add or remove based on their perspectives.

All the steps of the NGT process were repeated for each primary question: (1) What concerns me most when considering PrEP? (barriers), and (2) What is more likely to make me consider using PrEP? (facilitators). Participants were also informed about the underutilization of PrEP among BW in the US and encouraged to suggest ways to make PrEP more appealing to other BW. The facilitator ensured everyone had a chance to contribute.

### 2.4. Analytic Plan

Descriptive statistics were used to characterize the sample, analyzed with RStudio (2022.02.0 Build 443, Boston, MA, USA). Following NGT analysis recommendations [59], individual rankings were summed across all participants to derive the group-level rank order. Using the direct rating method, mean important scores and standard deviations (SD) were calculated for each of the 16 items across each rank question. The mean importance score, calculated by dividing the total points per item by the number of participants (n = 13), shows the group aggregate rank (with lower means representing higher importance), while the SD indicates the spread of agreement or disagreement around each item.

Qualitative analysis of the FG transcripts was conducted using NVivo 14 (Version 14; Lumivero, 2024, Denver, CO, USA) [80], following thematic coding of the rank responses and transcripts [20]. Two coders (AS and AG) performed thematic analysis to create major themes, sub-themes, and codes for results (i.e., specific barriers and facilitators related to PrEP uptake, along with suggested improvements).

Initially, one coder (AS) reviewed the first FG transcript to gain familiarity. An initial review of the raw data from NGT rankings (completed on Google Sheets) was also conducted to identify any anomalies or nuances within the data. Next, themes were developed in alignment with the overall FG questions. Line-by-line coding identified sub-themes and codes, forming a preliminary codebook. A second coder (AG) then reviewed the first (uncoded) FG transcript independently, using blind coding based on the preliminary codebook. Afterward, consensus coding was conducted, finalizing the codebook by resolving any disagreements in the codes. Once the codebook was finalized, each coder reviewed the transcript from the second FG using the finalized codebook. Findings were re-examined by both reviewers to ensure that the themes, sub-themes, and codes accurately represented the qualitative data and addressed any potential gaps. An independent auditor (JM), uninvolved in the FGs or coding, reviewed the themes, sub-themes, codes, and quotes for alignment and accuracy.

## 3. Results

### 3.1. Study Sample

Fourteen adult BW who were not living with HIV were enrolled in the study. Sample characteristics are displayed in Table 1. Participants’ mean age was 26 years (range: 19–44). Most participants self-identified as non-Hispanic (n = 13, 92.6%), heterosexual (n = 13, 92.6%), and were employed part-time and/or full-time (n = 9, 64.3%). Nearly half of the participants had earned a bachelor’s degree or higher (n = 6, 42.3%). Women lived in Florida (n = 4, 28.6%), Georgia (n = 4, 28.6%), Texas (n = 3, 21.4%), Delaware (n = 1, 7.10%), Louisiana (n = 1, 7.10%), and Maryland (n = 1, 7.10%). Over half of the women self-reported engaging in condomless vaginal sex with a male partner in the past 6 months (n = 9, 64.3%).

### 3.2. Key Concerns for Black Women When Considering PrEP

The ranked order of 16 items representing barriers of BW’s considerations to use PrEP are presented in Table 2. ‘PrEP side-effects’ (M = 3.85, SD = 2.65), followed by ‘How PrEP might affect other medicines’ (M = 4.31, SD = 4.10), having ‘No health insurance or not enough coverage’ to pay for PrEP (M = 4.92, SD = 4.23), ‘Being judged by my doctor’ (M = 5.77, SD = 4.24), and ‘PrEP is expensive’ (M = 3.47, SD = 3.27) were the top 5 most important items that concerned participants about potentially using PrEP. Table 3 describes the themes related to the items that BW identified as their highest and lowest concerns when considering PrEP use. During participant discussion of the top-ranked items, themes of PrEP side effects, Accessibility of PrEP; and Feeling judged emerged.

#### 3.2.1. Reasons for Top Concerns About PrEP

*PrEP side effects.* Participants described PrEP side effects as their top concern regarding their potential PrEP use. One woman learned about potential side effects of PrEP from a male friend who was taking it: *As I recall, he was experiencing a lot of lightheadedness and headaches, possibly nausea. He was throwing up sometimes. I’m not sure how long he took it for, if he ended up getting it changed. But I just remember that he had some effects of that nature* (23-year-old from Maryland, FG2). She specified that consulting with a doctor to discuss her specific concerns would be crucial to her deciding whether to use PrEP. Another woman described potential side effects as “*very, very scary*” due to their capacity to disrupt daily life: *Okay, so the side effects of drugs can actually be scary. Some might actually cause headache, vomiting. The side effects is always scary parts. Sometimes the person might feel tired or drowsy and you might be at a particular place where you’re not supposed to feel that way, maybe on the road or something that might cause you to faint. So, the side effect of drugs like this, it’s always very, very scary* (28-year-old from Georgia, FG1). During these conversations, several women specifically discussed the possible interaction between PrEP and other medications they are taking. For example, one woman said her number one concern would be: *a mix between the side effects and also how it could affect other medication…because I am on other medications. So that is the reason why I voted those two highest ranks* (20-year-old from Louisiana, FG1). She went on to say that these worries were compounded by PrEP “*being so new*.” Another woman said: *I agree with the PrEP side effects because…I figured that that would be the most important because somebody may be on medication already, you know what I mean? Like diabetes, high blood pressure or they may even have cancer, you know what I mean? And they want to make sure that the PrEP is not interfering with any of whatever other medication that they have going on because they can cause blood clot anywhere. It can cause a number of things. So, by that being the top priority is into, okay, “If I take this and I want to make sure that it is going to help me or is it going to interfere with what I already have going on?”* (34-year-old from Georgia, FG1).

*Accessibility of PrEP.* Cost and being able to obtain PrEP through one’s health insurance were also identified as the top concerns about accessing PrEP. One woman stated: *[PrEP is expensive] was my number one because I have a lot of expenses at hand and I wouldn’t want taking extra expenses on myself* (25-year-old from Florida, FG2). A younger participant described having to afford her own health insurance in the future as a personal concern; however, observing lack of insurance coverage among Black women made her choose ‘No health insurance or not enough coverage’ as a major concern: *…I’m not quite old enough to pay for my own health insurance, but I can just say based off of the people that are around me or my family members, that was a big concern, are things that might be useful to our communities aren’t always fully covered by insurance…* (19-year-old from Georgia, FG2). Another woman indicated concerns about obtaining PrEP through health insurance, given one’s employment status: *For the most part, I’ve always had insurance, but I was unemployed for five months and did not have insurance. That brought a lot of things to light about different things that I could afford and couldn’t afford. So that is a concern for me about getting on something like PrEP where you know you have to be consistent with it and making sure that I have coverage, especially how it interacts with other medications and making sure I have coverage consistently. That’s a concern for me* (44-year-old from Texas, FG2).

*Feeling judged.* Although ‘Being judged by my doctor’ was ranked as a top concern for women in their consideration to potentially use PrEP, the group widely discussed their health care provider as an important motivator for whether to choose to use PrEP. One woman described how race and gender concordance between the patient and doctor may help mitigate feeling judged, stating: *Black women are more comfortable talking a lot more with people of our same race and gender, and we feel less being judged by them while having conversations with them* (25-year-old from Florida, FG2). One woman spoke up and said, ‘Being judged by my doctor’ should be removed from the list of concerns: *…For me, I feel that being judged by my doctor should be removed from the list because, I mean, this PrEP, it’s coming from them. So, the idea of being judged by my doctor is questionable* (24-year-old from Florida, FG1). However, another woman raised concerns about how feeling religious judgment from a doctor jeopardizes the patient–provider relationship: *…Because of just unconscious bias and experiences I’ve had. But I’ve had the same race and gender, but there was still some judgment around, because of religious practices and things like that about sexual behaviors or things like that. So, there was still this, oh, well, you did this, and why are you doing that? So, it’s like, “I’m here to tell you what I need. You can keep the judgment.” So even though there was that same race and gender, there was still this sense of judgment there that still was uncomfortable to talk about other things that I may have needed. So even within that, sometimes it’s still hard* (44-year-old from Texas, FG2). Given that judgment from a doctor was discussed across both focus groups, women in this study did not speak much about feeling judged by family or friends. One woman said, “Feeling judged by family” and “Feeling judged by friends” should be combined as one item on the list (24-year-old from Florida, FG1). By contrast, family and friends were extensively described as important sources of support, suggesting judgment from their support system would not be a barrier among this group.

#### 3.2.2. Reasons for Lowest Concerns About PrEP

My partner may think I am cheating on them’ (M = 10.77, SD = 4.02), ‘No way to get to my doctor appointments’ (M = 11.46, SD = 3.27), ‘I do not trust PrEP’ (M = 11.54, SD = 3.31), ‘PrEP goes against my culture or religion’ (M = 11.92, SD = 4.52), and ‘No one to watch kids for my doctor’s visit’ (M = 12.46, SD = 3.05) were the least ranked items that concerned participants when considering PrEP (Table 2). During participant discussion of these least ranked items, themes of Already PrEP aware and Current lifestyle emerged (Table 3).

*Already PrEP aware.* When discussing the least ranked item, “I do not trust PrEP,” women did not speak much about their trust or distrust of PrEP in relation to other specific concerns, such as side effects of the medication. One woman stated, as it relates to the product in itself, “*I don’t really feel like it’s a trusting or a negative thing. I just think that there’s so many other things that are to be put into consideration when it comes to it, but nothing as it relates to trust*” (19-year-old from Georgia, FG2). However, women in this study may have already been aware and knowledgeable about PrEP prior to participating in this study. As one participant said, Black women are already aware of PrEP: *It may have something to do with where we are with PrEP. Maybe this group is already pre-educated on or knows someone or seen a commercial, versus maybe when it came out 10 years ago. There’s maybe a little bit of education there to say it’s been around. I’m familiar with it. This isn’t my first time hearing the word or the phrase. So, there’s not so much of, what is that? You don’t have to build that initial trust with this group. Maybe. That’s just a thought. For me, that’s the case. I’m pretty familiar with it. I’ve never used it, but I’m familiar with it in general.* (44-year-old from Texas, FG2).

*Current lifestyle: Parent* vs. *Not a parent.* Most participants stated not having any children as their reason for ranking ‘No one to watch kids for my doctor’s visit’ as their lowest concern: *I don’t have kids to take care of currently, so that’s why I listed it as the least* (23-year-old from Florida, FG2). Participants instead described having an established social support system to assist with child care if needed. One woman stated: *I don’t have kids, but if I did [I] have a good support system that would help me* (24-year-old from Florida, FG 1). Another woman stated, “*For me personally, me personally, that would not be one of my concerns just because I don’t have kids right now, but if I did have kids, I know my family would be willing to help with me, especially medically”* (20-year-old from Louisiana, FG1). One woman who did have children said, “*I think…no one to watch my kids…actually deserve to be ranked as the lowest because you definitely should have someone or even a friend or someone can help you take care of your kids*” (34-year-old from Georgia, FG 1). Another woman suggested using healthcare services that are available to assist individuals who have children and need child care: *If you can’t find someone to watch your kids for doctors visit, you can contact your health provider. There are actually some places that have policies or suggestions for managing appointment childcare. They might suggest you bring your kids along or virtual appointments like online appointments and all that* (24-year-old from Florida, FG1).

### 3.3. Key Factors Influencing Black Women’s Consideration to Use PrEP

The ranked order of 16 items representing facilitators of BW’s considerations to use PrEP are presented in Table 4. ‘Getting PrEP with a doctor of the same race’ (M = 5.23, SD = 3.53), ‘Getting PrEP with a doctor of the same gender’ (M = 6.23, SD = 4.85), ‘Receiving regular texts or emails reminding me to take PrEP’ (M = 7.38, SD = 3.65), and ‘Getting PrEP from my OBGYN as a part of routine sexual health services like Pap smears’ (M = 7.69, SD = 4.91) were the top 4 ranked PrEP facilitators by the BW. ‘Having PrEP mailed to me instead of picking it up in a pharmacy’ (M = 8.08, SD = 4.48) and ‘Assistance with PrEP costs’ (M = 8.08, SD = 4.03) were tied for the ranked 5th facilitator.

Table 5 describes the themes identified for the most important and least important facilitators for Black women when considering PrEP. During participant discussion of the top-ranked items, the theme of Providers of the same race and/or gender emerged.

#### 3.3.1. Reason for Top-Ranked Factors Influencing Black Women’s Consideration to Use PrEP

*Providers of the same race and/or gender.* Participants discussed the importance of having a healthcare provider who is the same race and/or gender as themselves: *At least for African American women… they’re a lot more relatable and they know a lot of my barriers, since they are of the same race. And it makes me feel more comfortable, especially having the same gender. I’ve had a male doctor before, but even the delivery of information, I like it preferably from a female, just from my past experiences* (23-year-old from Maryland, FG2). One woman described the importance of having a female doctor: *You feel like it’s going to be safe for you and sure that when discussing something like that with a person of my gender, I’m going to feel so comfortable to discuss anything [than] me discussing it with an opposite gender* (31-year-old from Texas, FG2); and another felt that seeing Black providers *reduces discrimination* (23-year-old from Florida, FG2). Among participants of FG1, there was disagreement. One woman described how preferences to see a female provider should not be ranked highly: *I feel that discussing PrEP with a doctor of the same gender, it’s really not that important. The issue of gender should not be considered when discussing PrEP because we are meant to believe that doctors are confidential, be its female or male…* (24-year-old from Delaware, FG1). However, other women in the FG reasserted the importance of having a provider that matches patient preferences: *I also think that with the gender [of the] doctor, I feel as though it should be a person’s preference. Maybe it should be worded a different way. Maybe, “Discussing PrEP with a doctor of your preference”* (34-year-old from Georgia, FG1). One woman shared her personal experience with unwanted advances during a healthcare visit with a male provider, which caused great discomfort: *I actually disagree with what she just said because I feel like for modesty and privacy, I feel uncomfortable, uncomfortable in talking with a male doctor because, especially when it’s related to my privacy, because I’ve actually done a checkup on my private area. Male doctor was checking me up, and they find things. After checking me up, he started asking for my digits, and he was like, “Okay, I need your contact and all that. I really like you”* (28-year-old from Georgia, FG1).

#### 3.3.2. Reasons for Lowest-Ranked Factors Influencing Black Women’s Consideration to Use PrEP

‘Using telehealth for all my PrEP follow-up appointments instead of in-person visits’ (M = 9.23, SD = 4.71), ‘Childcare during my PrEP appointments’ (M = 9.46, SD = 5.42), ‘Getting help from a professional to address concerns and experiences related to sexual health that could be distressing’ (M = 9.85, SD = 3.82), ‘Taxi voucher or Uber credit to help me get to my PrEP appointments’ (M = 10.77, SD = 3.53), and ‘Getting PrEP from a pharmacist without seeing any doctor’ (M = 11.31, SD = 4.36) were the least ranked facilitators by the BW (Table 4). Getting PrEP from a pharmacist is not necessary and Support for transportation emerged as primary themes (Table 5).

*Getting PrEP from a pharmacist is not necessary*. Pharmacists were not described as crucial to obtaining PrEP for BW in this sample. One woman said: *I ranked it the least because it something that concern my health in general so I won’t want to take medication without any prescription for my Doctor* (25-year-old from Florida, FG2). Another woman described direct-from-pharmacy services as not necessary, and suggested the item should be removed from the list of facilitators due to access of PrEP from community-based organizations: “*I want to say that getting PrEP from a pharmacist without seeing any doctor [should be removed], I feel as though if you go to a Planned Parenthood or a similar community organization, I feel as though that they would have resources already available. You know what I mean?*” (34-year-old from Georgia, FG1).

*Support for transportation.* Women in the sample expressed that transportation was not a major barrier towards their consideration of PrEP, highlighting the low ranking of ‘Taxi voucher or Uber credits to help [women] get to PrEP appointments.’ One woman stated that the taxi voucher or Uber credits were among the least important items to consider *because many of us have some type of support group that might be an assistance before choosing the taxi voucher or an Uber* (23-year-old from Maryland, FG2). However, another woman did express the need to still consider transportation even though it was ranked low: *Some people don’t have transportation or a lot of times when they do have transportation, for instance, like a bus or a car maybe they share with their spouse or their family members, they’re not able to go to certain doctors just because if they have insurance, some of their insurance will allow them to go to certain providers and majority of the time those providers are actually nowhere near where they’re actually located. So, I think you should definitely take in consideration, of course insurance but also transportation access* (20-year-old from Louisiana, FG1).

### 3.4. Strategies to Enhance PrEP Appeal Among Black Women

**Figure 2:**
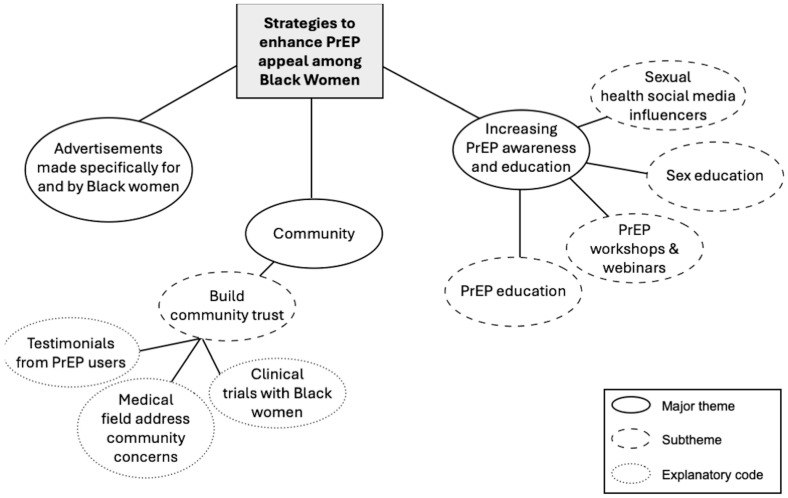
Strategies to enhance PrEP appeal among Black women.

In each FG session, participants were asked to offer ideas about what strategies may work to enhance the appeal of PrEP to other BW. The themes, sub-themes, and explanatory codes about these strategies are shown in Figure 2.

*Advertisements made specifically for and by Black women*. Creating advertisements promoting PrEP to BW was a strategy widely discussed across both FGs. Participants wanted to see BW in the advertisements and wanted the advertisements to communicate crucial information about PrEP, including its benefits to BW, specifically: *I think that when it comes to advertising, especially advertising how it benefits Black women specifically, a lot of times the benefits are very general. So, it might be how it might affect everyone or the benefits that it might have on just the general population, but specifying how it can specifically benefit African American women in the situations or circumstances that we experience* (19-year-old from Georgia, FG2). One woman stated: …*I would like to see Black women doing the advertising* (23-year-old from Florida, FG2). Another woman felt that the advertisements could expand beyond the benefits of PrEP as well: *…you always can expand on not only just the benefits, but where you can go and purchase, the different flexibilities and the availability of getting it* (23-year-old from Maryland, FG2).

*Community.* Participants also spoke extensively about strategies that prioritize the concerns and needs of BW. These strategies included efforts to build community trust: *Really, just creating that trusted community, which I feel like it’s really needed to get, especially just the Black community in general, to take medication. Just because, due to a lot of the lack of trust in the medical field. So, yeah. Because I know my mom, personally, she’s not really for medicine-taking especially over-the-counter. So, just making that trust and having that knowledge* (20-year-old from Louisiana, FG1). Engaging community leaders was suggested by another woman: *I would just say that engaging community leaders…and the AIDS care providers who are trusted within the Black communities can help spread the awareness and address concern or misconceptions* (31-year-old from Texas, FG2). To increase community trust towards PrEP, one woman suggested having testimonials from PrEP users in the community: *So, what would make it more interesting is actually hearing the testimonies of how people that have used PrEP and how it has changed their life. Has it made them feel more safe or made them feel more, not safe, but more prepared?* (20-year-old from Louisiana, FG1). Another woman concurred: *I agree…I honestly feel as though it should be, in order for we, as Black women to have some form of interest in it, there needs to definitely be integrity, far as if they have already had it. To share their experience through maybe a video, a review, or something in that nature. To where they can let it be known that, “Hey, I have taken it. I’ve taken it over a month. This is how I feel.”* (34-year-old from Georgia, FG1). Someone else wanted to see success stories from BW: *Only one last thing is always success stories work. If somebody can do a journey map of how they got it and how it helped them and how it’s going. Just to add that from a Black woman’s perspective...Yes. Successfully. Like, maybe they were hesitant and like, “Oh, I didn’t think it was for me, but like, oh, I take it and this is how I talk to my partner,” and show they’re actually doing it and taking it* (44-year-old from Texas, FG2).

*Increasing PrEP awareness and education.* Participants described PrEP awareness and education among BW as necessary to improve interest in PrEP: *Education is empowering, so kind of taking the onus on ourselves as we do with heart health and everything else to say, “This is something that we can do for ourselves and let’s empower each other to, there’s something that we can do.” We’re not just sitting back and letting the numbers increase. We can actually do something and this is something that we can do and here’s how* (44-year-old from Texas, FG2). One woman felt that *awareness among Black women could be fostered through educating PrEP importance* (24-year-old from Georgia, FG2), and another woman mentioned that this education should start early: *[There] should be PrEP awareness for young Black girls who will later turn into a full-grown adults* (25-year-old from Florida, FG2).

Several modalities were discussed, including workshops and webinars that can focus on testimonials from BW. The women also described normalizing PrEP by adding PrEP education to sexual education curricula: *I was thinking along the lines of sex education, how there’s a lot of talk about contraception, and safe sex, and all that. So, add PrEP to the mix so that it becomes something that’s more normalized, and not something that’s not well-known* (24-year-old from Florida, FG1). Lastly, BW in this study mentioned partnering with sexual health social media influencers to promote PrEP to BW by having conversations about dating, and reaching a wider audience of BW:

*…social media influencers are having these conversations about relationships and things like that in the Black community, they’re doing all these going around and doing tours, they rarely talk about sexual health, let alone protection and how we can reduce the rates of HIV and take the ownership of ourselves. But if PrEP can be part of the conversation and part of the agenda, so talking to the influencers of these events that talk about Black relationships and dating. If some kind of way PrEP can be brought up in a positive way, not always about the gloom and doom, but like, “Hey, this is something we can do y’all.” It’s a positive way as we’re talking about dating* (44-year-old from Texas, FG2).

*One thing that I know that they’re starting to do now, especially with social media influence, I don’t know if you all see on TikTok how they have people coming on promoting, I think it’s Planned Parenthood or some brand that they have partnered with specifically influencers to get them to incorporate basically their product in everyday conversations and everyday talks. I think that that’s a very smart way, especially if you want to get younger generations involved and onto it, younger Black women, just making it more in their face and incorporated in the things that they see on a daily basis* (19-year-old from Georgia, FG2).

## 4. Discussion

The current study used NGT to examine how and why BW prioritize critical barriers and facilitators for PrEP use. Our findings offer insight into high-priority barriers that need addressing and key facilitators that could enhance PrEP uptake among BW. Multifaceted approaches that prioritize PrEP education on PrEP side effects, improve accessibility regarding cost and insurance coverage, and improve the patient–provider relationship are needed to effectively address the PrEP needs of BW.

### 4.1. PrEP Side Effects and Medication Interactions

Participants expressed concerns about potential side effects and interactions between PrEP and other medications. This worry is commonly cited in the literature [16,19]. For example, a systematic review by Willie and Dale [19] identified twenty-four studies where PrEP side effects were a recurring concern among BW [19], with three studies mentioning medication interaction [7,65,76]. Managing multiple medications can be challenging for those considering PrEP, yet most research on drug interactions has focused on sexual minority men and transgender women [17,35], with limited studies involving BW in the US [81,82,83,84]. We lack a clear understanding of PrEP literacy among BW and how it influences their perception of PrEP. Clinical research is needed to explore how PrEP side effects impact BW and to identify the best strategies for managing these side effects. Further research should also determine the best methods for delivering the specific PrEP information that BW prefer and need.

### 4.2. Need to Improve PrEP Access by Addressing Cost and Insurance Coverage

Lack of health insurance or inadequate coverage was a major concern for participants considering PrEP, while financial support was seen as a facilitator. The perceived financial burden of PrEP has been cited in other studies [19,24,47,76,84], such that the lack of healthcare insurance coverage and healthcare access have been identified as major barriers to PrEP engagement among populations vulnerable to acquiring HIV [85,86,87]. Our research suggests these financial and systemic barriers hinder PrEP engagement among BW, highlighting the need for targeted interventions. Chan et al. [88] found that many individuals on PrEP were more likely to pay for PrEP using commercial or private insurance rather than publicly sponsored programs. Expanding healthcare coverage and offering financial support programs could significantly enhance PrEP uptake and reduce the HIV prevention gaps for BW. However, most BW in this study live in Southern states without Medicaid expansion (i.e., Florida, Georgia, Texas) [1], limiting their access to preventative services [89,90]. A review of state policies found that nearly half of the 20 states with the highest HIV incidence among cisgender women have not expanded Medicaid [91], resulting in higher uninsured rates and increased pressure on drug assistance programs to cover PrEP costs [92]. For women with limited financial resources, this creates a major access issue. Addressing this disparity will require Medicaid expansion, robust drug assistance programs, and initiatives to lower PrEP-related clinical costs. Without these changes, access barriers for BW in the South will persist, perpetuating inequities in HIV prevention.

### 4.3. The Patient–Provider Relationship and PrEP

Throughout the focus groups, women discussed their healthcare providers as important motivators for considering PrEP, specifically when there was racial and gender concordance. Findings suggest that race and gender matching between patient and provider can help reduce medical mistrust and perceived stigma or expectations of being judged by providers. Studies have shown that such concordance improves health outcomes for women [84,93,94,95,96,97]. For example, Hill et al. [84] conducted focus groups among 20 BW to explore how social and structural constructs influence individual decisions to use PrEP. Their study participants preferred a healthcare provider of the same race and gender because it contributed to their comfort with self-advocating for their health [85]. Townes et al. [96] found that BW preferred providers who shared their race and gender, as this created a sense of connection and understanding in their sexual health care experiences. However, not all women in the current study felt that race and gender concordance were important. Future PrEP programs and interventions ought to consider offering BW different provider options to accommodate their own perceived preferences, which could help improve the connection they have with the provider, bolster communication about PrEP, and address possible medical mistrust [43,45,98,99].

An obstetrician-gynecologist (OBGYN) may be an ideal provider for PrEP information, screening, and prescription for BW [100,101]. In this study, participants ranked ‘getting PrEP from an OBGYN as a part of routine sexual health services’ as an important facilitator, more so than receiving PrEP from a pharmacist or community organization. Previous literature has also found that, if offered, BW may prefer getting PrEP from their OBGYN [77,78,100,102,103]. In line with prior research, our findings may reflect BWs’ desire for PrEP to be integrated into the sexual and reproductive healthcare they already access. Women typically visit their OBGYN annually for exams that cover reproductive health, contraception, and wellness, often including discussions about sexuality and sexual history. As such, women may feel comfortable discussing sexual health with providers whose backgrounds are specific to women’s sexual and reproductive health. Extending PrEP delivery among clinicians across disciplines, like an OBGYN, may help improve access and uptake of PrEP among women seeking different types of healthcare services, thus creating a more comprehensive and patient-centered approach to HIV prevention.

### 4.4. Social Support Systems: Familial Support and Peer Support

Unlike previous research, which has indicated that lack of social support and fear of stigma are key factors related to lack of PrEP uptake among HIV vulnerable populations [40,41,104], none of the women in this study described feelings of stigma, discrimination, or shame related to their possible PrEP use stemming from family or friends. For instance, in our sample, due to familial support or support from friends, participants ranked items that focused on needing child care and lack of transportation as low concerns regarding their consideration to use PrEP. It is possible that these barriers are not relevant to younger women or women without children, or that women in this sample do not foresee issues stemming from social support. Our findings indicate the need to understand how support systems influence PrEP uptake when not directly tied to PrEP (e.g., transportation, childcare, financial support, gender norms, etc.). BW may not realize the kinds of support they may need until they are actually using PrEP, dealing with the medical system to schedule appointments, or interacting with sexual partners while on PrEP. Future studies should consider exploring how social support systems can influence PrEP uptake among BW, particularly how family dynamics or responsibility may impede the use of PrEP among certain demographics of BW.

### 4.5. Strategies to Enhance PrEP Appeal Among Black Women

Women in this study identified three primary strategies to increase PrEP appeal among other BW: (1) ads made specifically for and by BW; (2) building community trust through testimonials from PrEP users, including providing information about PrEP clinical trials among BW; and (3) raising PrEP awareness and education through social media, workshops, webinars, and sexual educators. These strategies align with previous research and have been successfully used to increase PrEP appeal among BW [19,77,105]. For example, a BW and PrEP (BWAP) Task Force highlighted the effectiveness of social media campaigns and provider education initiatives to address PrEP barriers in Louisiana [81]. Kudrati et al. [105] found that social media is a promising approach for generating PrEP awareness due to its reach, accessibility, affordability, and usability. PrEP education workshops also hold promise. Chandler et al. [106] developed a culturally relevant PrEP program for Black college women, who reported increased understanding and likelihood of PrEP use. Community trust, fostered through testimonials and peer endorsements, can counteract stigma and medical mistrust [107], which are common barriers to PrEP uptake among BW. Overall, these strategies highlight the need for tailored community-centered interventions to support BW in accessing and using PrEP as a vital HIV prevention tool.

### 4.6. Limitations

Limitations of this study are important to acknowledge. First, only BW from the US South who met specific eligibility criteria were included in the study, which limits the generalizability of the findings. However, the study sample did represent BW across a diverse range of socioeconomic statuses regarding education, income, and employment status. Second, it is possible that having a larger sample of BW in this study may have produced different barrier and facilitator rankings and related findings. Although the literature does not specify an appropriate sample size for NGT, this study’s sample size aligns with the small number of FGs used in prior studies [108,109,110]. Third, the 90-minute timeframe allotted for focus groups may have limited the depth of discussion about ranked items. Fourth, the NGT step for generating ideas was not included in this study, and instead included only predefined PrEP barriers and facilitators. Despite drawing from the extant literature, other relevant barriers and facilitators related to the potential uptake of PrEP among BW could exist that were not included in this study. Nonetheless, BW were asked to provide additional items to add to the list of barriers and facilitators presented, allowing them to identify factors not captured in prior research. Also, only the highest and lowest priority items were discussed in focus groups, possibly overlooking other significant items. Fifth, unlike traditional NGT, participants were not prompted to change their rankings after discussions, which may have missed shifts in opinions or deeper insights. Lastly, we did not collect extensive demographic data on interpersonal factors (i.e., children, primary transportation), which could have provided further context for ranking decisions. Notwithstanding these limitations, our study successfully identified how and why BW prioritize barriers and facilitators in considering PrEP use.

## 5. Conclusions

This study is among the first to apply Nominal Group Technique (NGT), a consensus technique, in a web-based focus group setting among a population that is demographically and geographically underrepresented among PrEP users and within PrEP-related research. Our findings underscore the need for multifaceted approaches that prioritize PrEP education, accessibility, and the patient–provider relationship, specifically race and gender concordance, to effectively address the PrEP needs of BW. These findings also highlight how a novel application of NGT through a web-based format can be useful in its effectiveness in eliciting preferences about PrEP from BW. To create an impactful and patient-centered PrEP program or intervention for BW living in the Southern US, it is imperative to identify modifiable targets to improve PrEP uptake, including how these targets are prioritized in comparison to one another. Interventions that are designed to address the most salient barriers and leverage the most impactful facilitators are better positioned to increase the likelihood of successful PrEP engagement, uptake, and adherence among BW vulnerable to HIV acquisition.

## Figures and Tables

**Table 1:** Sociodemographic characteristics of Black cisgender women (N = 14).

Sociodemographic Characteristics	N (%)
Age: M (SD) [range: 19–44]	26 (6.2)
Ethnicity: Non-Hispanic	13 (92.9)
Employment status: Employed	9 (64.3)
Education: Had a Bachelor’s or higher degree	6 (42.3)
Household income: >$35,000	8 (57.1)
Sexual orientation: Heterosexual	13 (92.9)
Engagement in HIV vulnerable behavior(s) in the past 6 months ^a^	
* Condomless vaginal sex with a male partner*	9 (64.3)
* Condomless anal sex with a male partner*	2 (14.3)
* Diagnosed with an STI*	3 (21.4)
* Sex exchange*	3 (21.4)
* Did not engage in any HIV vulnerable behaviors*	4 (28.6)
Engaged in more than 1 HIV vulnerable behavior in the past 6 months	5 (35.7)
Residential State	
* Delaware*	1 (7.1)
* Florida*	4 (28.6)
* Georgia*	4 (28.6)
* Louisiana*	1 (7.1)
* Maryland*	1 (7.1)
* Texas*	3 (21.4)

^a^ Respondents could select more than one response.

**Table 2:** Barriers related to potential PrEP use among Black cisgender women (N = 13).

What Concerns Me Most When Considering PrEP?	Ranking	Total (Sum) Score	Mean Importance Score	Standard Deviation
PrEP side-effects	1	50	3.85	2.65
How PrEP might affect other medicines I am taking (e.g., birth control or diabetes pills)	2	56	4.31	4.10
No health insurance or not enough coverage	3	64	4.92	4.23
Being judged by my doctor	4	75	5.77	4.24
PrEP is expensive	5	84	6.46	3.47
Keeping my use of PrEP private	6	87	6.69	3.86
Being judged by my family	7	124	9.54	3.00
Being judged by my friends	7	124	9.54	3.03
I am not sure PrEP works well	9	126	9.69	4.58
I am thinking about getting pregnant soon	10	128	9.85	3.74
Having to discuss my sexual health with the doctor	11	129	9.92	4.71
My partner may think I am cheating on them	12	140	10.77	4.02
No way to get to doctor appointments	13	149	11.46	3.27
I do not trust PrEP	14	150	11.54	3.31
PrEP goes against my culture or religion	15	155	11.92	4.52
No one to watch kids for my doctor’s visit	16	162	12.46	3.05

*Notes*. Due to a technical error, one participant could not provide a rank score, so the NGT results represent the rankings from 13 participants. This participant still contributed to the group discussions.

**Table 3:** Themes derived from qualitative analysis of ranked barriers related to PrEP.

What Concerns Me Most When Considering PrEP?		
NGT Ranked Items	Themes	Definition of Themes
**Top concerns about using PrEP**		
PrEP side effects	PrEP side effects	Concerns and uncertainty about the side effects of taking PrEP
How PrEP might affect other medicines I am taking (e.g., birth control or diabetes pills)	PrEP side effects	Interaction with other medications
No health insurance or not enough coverage	Accessibility of PrEP	Concerns about health insurance covering PrEP
Being judged by my doctor	Feeling judged	Feeling judged for requesting/taking PrEP
PrEP is expensive	Accessibility of PrEP	Concerns about the cost of PrEP
**Lowest concerns about using PrEP**		
I do not trust PrEP	Already PrEP aware	Black women are already aware of PrEP
No one to watch the kids for my doctor’s visit	Current lifestyle	Parent vs. Not a parent—Having or Not having children

*Notes*. NGT: Nominal Group Technique. The table only displays items discussed during focus group discussions and thus identified in the thematic analysis.

**Table 4:** Facilitators related to potential PrEP use among Black cisgender women (N = 13).

What Is More Likely to Make Me Consider Using PrEP?	Ranking	Total (Sum) Score	Mean Importance Score	Standard Deviation
Discussing PrEP with a doctor of the same race	1	68	5.23	3.53
Discussing PrEP with a doctor of the same gender	2	81	6.23	4.85
Receiving regular texts or emails reminding me to take PrEP	3	96	7.38	3.65
Getting PrEP from my OBGYN as a part of routine sexual health services, like Pap smears	4	100	7.69	4.91
Having PrEP mailed to me instead of picking it up in a pharmacy	5	105	8.08	4.48
Assistance with PrEP costs	5	105	8.08	4.03
Getting help to discuss PrEP with my partner	7	106	8.15	4.64
Belonging to a group of Black women using PrEP for questions and support	8	107	8.23	4.30
Having someone you can call or text for help with insurance, appointments, and PrEP support	9	108	8.31	2.87
Using an app for medical consultations, tests, and home delivery of PrEP	10	110	8.46	5.67
Getting PrEP from Planned Parenthood or a similar community organization	11	112	8.62	4.34
Using telehealth for all my PrEP follow-up appointments instead of in-person visits	12	120	9.23	4.71
Childcare during my PrEP appointments	13	123	9.46	5.42
Getting help from a professional to address concerns and experiences related to sexual health that could be distressing	14	128	9.85	3.82
Taxi voucher or Uber credit to help me get to my PrEP appointments	15	140	10.77	3.53
Getting PrEP from a pharmacist without seeing a doctor	16	147	11.31	4.36

*Notes*. Due to a technical error, one participant could not provide a rank score, so the NGT results represent the rankings from 13 participants. This participant still contributed to the group discussions.

**Table 5:** Themes derived from qualitative analysis of ranked facilitators related to PrEP.

What Is More Likely to Make Me Consider Using PrEP?		
NGT Ranked Items	Themes	Definition of Themes
**Most likely to influence PrEP use**		
Discussing PrEP with a doctor of the same race	Providers of the same race and/or gender	Race/gender congruency between Black women and PrEP providers (healthcare professionals)
Discussing PrEP with a doctor of the same gender	Providers of the same race and/or race	Race/gender congruency between Black women and PrEP providers (healthcare professionals)
**Least likely to influence PrEP use**		
Getting PrEP from a pharmacist without seeing a doctor	Getting PrEP from a pharmacist is not necessary	A pharmacist is not necessary because of access to primary HCP or other sexual/reproductive healthcare services
Taxi voucher or Uber credit to help me get to my PrEP appointments	Support for transportation	Transportation is not a major barrier

*Notes*. NGT: Nominal group technique. The table only displays items discussed during focus group discussions and thus identified in the thematic analysis.

## Data Availability

The qualitative data generated and analyzed during the study are not publicly available because they contain information that could compromise participant privacy and consent. The corresponding author can be contacted for follow-up questions or concerns.

## Supplementary Materials

The following supporting information can be downloaded at: https://www.mdpi.com/article/10.3390/ijerph23050571/s1, Consolidated Semi-Structured Focus Group Discussion Guide.

## Abbreviations

The following abbreviations are used in this manuscript:

BW	Black women
FG	Focus group
HIV	Human Immunodeficiency Virus
NGT	Nominal Group Technique
PrEP	Pre-exposure prophylaxis
