# Supplementary material for: Prioritizing Barriers and Facilitators to PrEP Uptake Among Black Cisgender Women: Key Factors Identified Through Nominal Group Technique
Source: Int J Environ Res Public Health. 2026 Apr 28;23(5):571. doi: 10.3390/ijerph23050571 (PMC13206111; doi:10.3390/ijerph23050571)
Supplement: Supplementary file 1 [file ijerph-23-00571-s001.zip › ijerph-4237997-supplementary.pdf]

**Introduction**

Welcome, overview of focus group objectives, and setting of ground rules.

- PowerPoint presentation provided to present necessary PrEP information and NGT process.
- All relevant terms (e.g., "PrEP") provided, and all participants given the opportunity to ask any questions about any information provided before participants begin ranking.
- Remind participants about confidentiality, assure them there are no "wrong answers," and maintain a respectful atmosphere - especially since this involves sexual health topics.

**Rankings**

Participants rank 16 response options for question #1: What concerns me most when considering PrEP?

- Participants ranked all items simultaneously (e.g., 1 to 16)
- Offer participants the chance to adjust their rankings after the initial ranking.

**Discussion** (For any possible questions that invite a participant to elaborate, ensure all questions invite differing opinions (e.g., Who has a different perspective? Or Does anyone have a different perspective?)

*Looking at the rankings, we see that most of the group chose [item that was chosen as #1] as the item that concerns you all the most when considering PrEP and [item that has the highest number] as the item that concerns the group the least when considering PrEP.*

*Sub Question 1: Why do you think [item that was chosen as #1] was chosen as the item that concerned you the most?*

*- Do you agree or disagree with the item that was chosen as #1? Why?*
** Same question for top 3 choices*

*Sub Question 2: Why do you think [item that has the highest number] was chosen as the item that concerned you the least (i.e., highest number)?*

*- Do you agree or disagree with the item that was chosen as #16? Why?*
** Same question for bottom 3 choices*

*Sub Question 3: What items are currently not listed that you think should be added? Why?*

*Sub Question 4: Which items do you think should be removed from this list? Why?*

*Sub Question 5: Which items were confusing or hard to understand? Why?*

- *How would you rephrase these items?*

*For any possible questions that invite a participant to elaborate, ensure all questions invite differing opinions.*

**Rankings**

Participants rank 16 response options for question # 2: What is more likely to make you consider using PrEP?

- Participants ranked all items simultaneously (e.g., 1 to 16)
- Offer participants the chance to adjust their rankings after the initial ranking.

**Discussion** (For any possible questions that invite a participant to elaborate, ensure all questions invite differing opinions (e.g., Who has a different perspective? Or Does anyone have a different perspective?)

*Looking at the rankings, we see that most of the group chose [item that was chosen as #1] as the item that is more likely to make you consider using PrEP, and [item that has the highest number] as the item that is least likely to make you consider using PrEP.*

*Sub Question 1: Why do you think [item that was chosen as #1] was chosen as the item that is more likely to make you consider using PrEP?*

*- Do you agree or disagree with the item that was chosen as #1? Why?*
** Same question for top 3 choices*

*Sub Question 2: - Why do you think [item that has the highest number] was chosen as the item that is least likely to make you consider using PrEP?*
*- Do you agree or disagree with the item that was chosen as #16? Why?*
** Same question for bottom 3 choices*

*Sub Question 3: What items are currently not listed that you think should be added? Why?*

*Sub Question 4: Which items do you think should be removed from this list? Why?*

*Sub Question 5: Which items were confusing or hard to understand? Why?*

- *How would you rephrase these items?*

**Final Question**

According to the Centers for Disease Control and Prevention, compared to women of other races and ethnicities, Black women in the US are not using PrEP. Can you share ideas to make PrEP more interesting for your friends or other Black women?

*Prompt:* What are some ideas for making PrEP more appealing?
